# Regulation of epidermal barrier function and pathogenesis of psoriasis by serine protease inhibitors

**DOI:** 10.3389/fimmu.2024.1498067

**Published:** 2024-12-16

**Authors:** Juanjuan Wang, Junqin Li, Ling Zhou, Hui Hou, Kaiming Zhang

**Affiliations:** ^1^ Shanxi Key Laboratory of Stem Cells for Immunological Dermatosis, Institute of Dermatology, Taiyuan Central Hospital, Taiyuan, China; ^2^ State Key Breeding Laboratory of Stem Cells for Immunological Dermatosis, Institute of Dermatology, Taiyuan Central Hospital, Taiyuan, China

**Keywords:** serine protease inhibitors, psoriasis, inflammation, skin barrier function, hyper-proliferation

## Abstract

Serine protease inhibitors (Serpins) are a protein superfamily of protease inhibitors that are thought to play a role in the regulation of inflammation, immunity, tumorigenesis, coagulation, blood pressure and cancer metastasis. Serpins is enriched in the skin and play a vital role in modulating the epidermal barrier and maintaining skin homeostasis. Psoriasis is a chronic inflammatory immune-mediated skin disease. At present, most serpins focus on the pathogenesis of psoriasis vulgaris. Only a small number, such as the mutation of SerpinA1/A3/B3, are involved in the pathogenesis of GPP. SerpinA12 and SerpinG1 are significantly elevated in the serum of patients with psoriatic arthritis, but their specific mechanism of action in psoriatic arthritis has not been reported. Some Serpins, including SerpinA12, SerpinB2/B3/B7, play multiple roles in skin barrier function and pathogenesis of psoriasis. The decrease in the expression of SerpinA12, SerpinB7 deficiency and increase in expression of SerpinB3/4 in the skin can promote inflammation and poor differentiation of keratinocyte, with damaged skin barrier. Pso p27, derived from SerpinB3/B4, is an autoantigen that can enhance immune response in psoriasis. SerpinB2 plays a role in maintaining epidermal barrier integrity and inhibiting keratinocyte proliferation. Here we briefly introduce the structure, functional characteristics, expression and distribution of serpins in skin and focus on the regulation of serpins in the epidermal barrier function and the pathogenic role of serpins in psoriasis.

## 1 Background

Serine protease inhibitors (Serpins) is a new family proposed by Hunt et al. (1, 2) in 1980 on the basis of the conserved primary structure of ovalbumin, α1 antitrypsin, and human antithrombin. Many members of this family can inhibit the activity of serine proteases such as chymotrypsin, so they are named serine protease inhibitors (Serpins) (3, 4). Currently, approximately 1500 serpin sequences have been identified in animals, poxvirus, plants, bacteria and archaea (5, 6). The Serpins family contains a group of structurally similar but functionally distinct proteins, making it the largest and most functionally diverse family of protease inhibitors. Serpins are involved in inflammation, immunity, tumorigenesis, blood coagulation, blood pressure and cancer metastasis (7).

Psoriasis is a chronic, recurrent and inflammatory immune-mediated skin disease induced by genetic and environmental interaction (8). Psoriasis is characterized by excessive proliferation and poor differentiation of keratinocytes, immune cell infiltration, skin inflammation as well as impaired epidermal barrier (8–10). According to the clinical characteristics of psoriasis, psoriasis can be divided into: psoriasis vulgaris, psoriasis arthropathica, psoriasis erythrodermic and psoriasis pustulosa (11), of which vulgaris is the most common type, and other types are mostly converted from psoriasis vulgaris (12, 13). Pustular psoriasis can be further divided into localized (e.g. palmoplantar pustulosis) and generalized pustular psoriasis (GPP) (14). In the skin, the epidermis is an abundant source of proteases and protease inhibitors (15). Endogenous and exogenous proteases, such as caspases, cathepsins, kallikreins and proteases from microorganisms, play important roles in the desquamation and defense regulation of the stratum corneum. Protease inhibitors contribute to skin integrity and protective barrier function by regulating their proteolytic activity (16). An imbalance of proteases/protease inhibitors in the skin can lead to changes in protease hydrolysis of target proteins such as filaginins, cytokines, and receptors, inducing skin inflammatory responses and barrier dysfunction (16). Serpins are the most diverse superfamily of protease inhibitors (1). Serpins are abundant in skin and play an important role in maintaining skin homeostasis. In recent years, a growing body of literature have shown that some serpins are differentially expressed genes in psoriasis lesions (17, 18), gene polymorphisms and mutations of some serpins are also involved in psoriasis pathogenesis (19, 20). These genetic changes in serpins lead to changes in their target protease activity and proteolysis of target proteins, which contribute to psoriatic inflammation and skin barrier dysfunction. Here we briefly introduce the structure, functional characteristics, expression and distribution of serpins in skin and focus on the regulation of serpins in the epidermal barrier and the pathogenic role of serpins in psoriasis.

## 2 Structural, functional characteristics of serpins in skin

**Figure 1:**
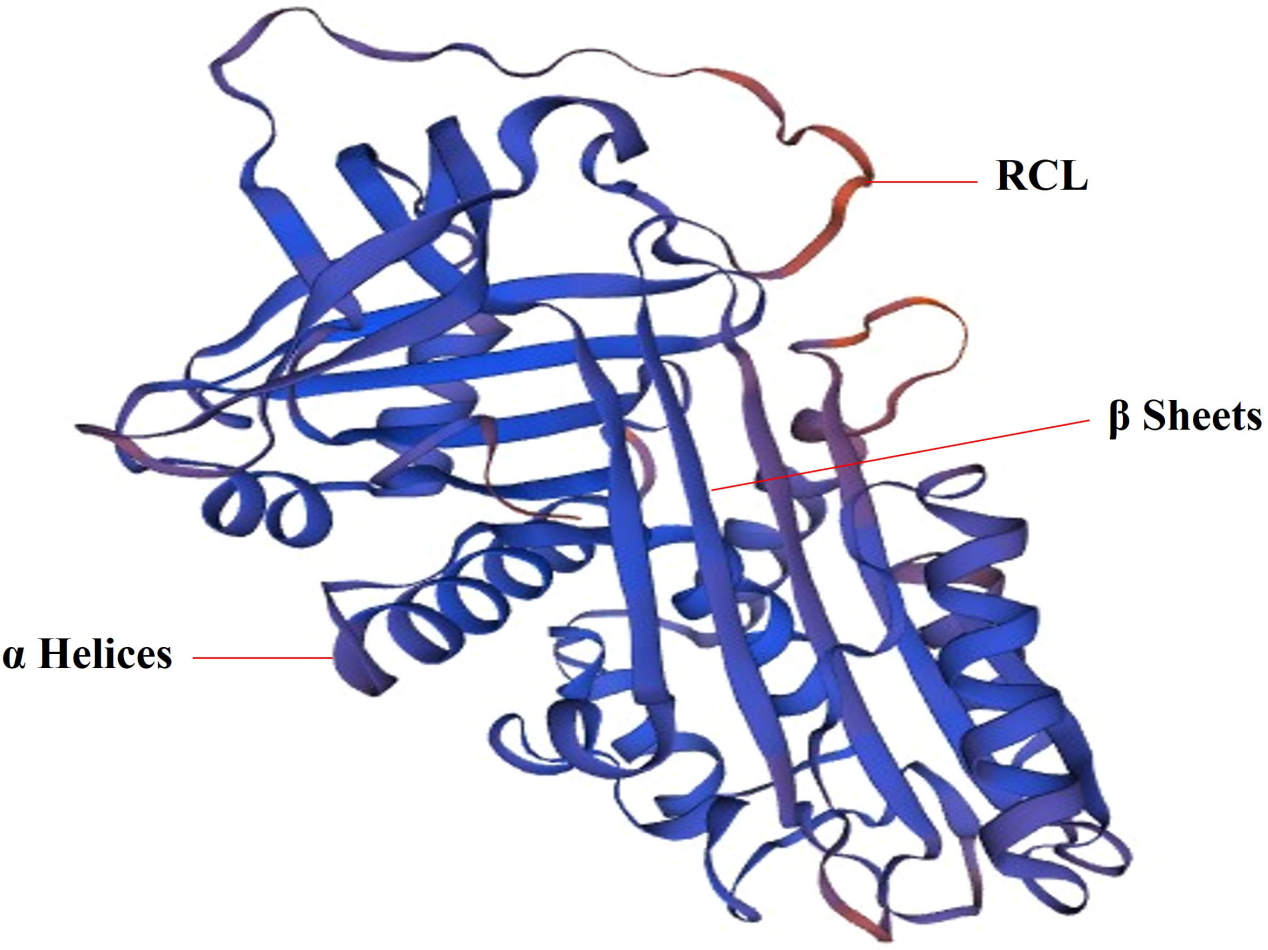
The 3D structure of SerpinA1. SERPINA1 with labeled structural elements: α helices, β sheet and reactive center loop (RCL).

Serpin is a single chain protein, consisting of 350 ~ 400 amino acids. The serpins have a core structure of about 380 amino acids, which folds into a very conservative, typical three-dimensional structure in a metastable state. This structure consists of 8 ~ 9 α helices (hA ~ hI) and 3 β sheets (A-β, B-β, C-β) (21) (**Figure 1**). The region of interaction with the target enzyme is a bare ring motif, also known as the reaction center ring (RCL) (**Figure 1**), which is spread on the serpin scaffold and contains the protease recognition site P1 (5). The Serpin protein superfamily is divided into groups called clades based on their sequence similarity. Clades are classified into A-P, and human serpins are phylogenetically classified into clades A-I (21, 22). In the human genome, there are 36 protein-coding genes of serpins (21) and the two largest clades of the 36 identified serpins are extracellular “clade A” (12 members located on chromosomes 14, and X) and intracellular “clade B” (13 members located on chromosomes 6 and 18) (22–24). Clade C (serpinC1) is located on chromosome 1. Clade D (serpinD1) is located on chromosome 22. Clade E (3 members) are located on chromosome 2, 7 and 13. Clade F (2 members) are located on chromosome 17. Clade G (serpinG1) and Clade H (serpinH1) are both located on chromosome 11. Clade I (2 members) are located on chromosome 3.

Although Serpins have similar structural characteristics, their modes of action are very different. According to these characteristics, Serpin superfamily members are divided into inhibitory and non-inhibitory serpins. The inhibitory serpins can suppress the serine proteases, caspases and papain-like cysteine proteases by an irreversible suicidal mechanism (21, 25, 26) and we summarized the target enzymes inhibited by each human serpin member in **Table 1**. However, the non-inhibitory serpins exhibit functions unrelated to inhibiting catalytic activity, such as hormone transport or blood pressure regulation (5, 6, 21, 107). For example, SerpinA8 is a blood pressure regulator (108). SerpinA6 and A7 are responsible for the transport of cortisol (109) and thyroid hormone (46), respectively. In humans, most (24 out of 36) serpins are inhibitory (**Table 1**). The inhibitory serpins plays an important role both intracellular and extracellular, including coagulation regulation (110), inflammatory response, complement activation (102) and immune regulation. A number of serpins play vital roles in the inflammation and immunity. In Clade A, there are some inhibitory serpins containing anti-inflammatory molecules (SerpinA1, A3, A4, A12) (111–115) and non-inhibitory serpins containing inflammatory molecule(SerpinA6, 8) (20, 47, 109, 170). The inhibitory SerpinA9 is involved in maturation and maintenance of naïve B cells (50). In Clade B, there are some inhibitory serpins containing anti-inflammatory molecules (SerpinB1, B2, B6) (116, 117) and pro-inflammatory molecules (SerpinB3/B4, B10) (78, 118) (**Table 1**). Act as potent inhibitors of proteases in neutrophils, SerpinA1 (21), A3 (33, 34), B1 (116) and B6 (80) are thought to protect monocytes, neutrophils, and onlookers from ectopic neutrophil-derived proteases during inflammation (82, 119). SerpinB1 is a protective immunomodulatory that protects the host’s antimicrobial defenses and prevents tissue damage (61, 120). SerpinB9 is a potent inhibitor of granzyme B, which is located in the same subcellular compartment of cytotoxic lymphocytes as granzyme B, and can protect cytotoxic lymphocytes from the destruction of granzyme B (89). SerpinB9 also inhibits IL-1β maturation by inhibiting Caspase-1. Mutations in the SerpinB9 gene may contribute to the development of autoinflammatory diseases (121). Some serpins are important hemostatic regulators, including SerpinA5 (42), SerpinA10 (51), SerpinC1 (98), SerpinD1(heparin cofactor II) (122), SerpinE1(PAI1) (122), SerpinE2 (100) and SerpinF2 (122). SerpinC1 not only has anticoagulant and anti-inflammatory effects (98), but also has the effect of inhibiting liver cancer (123) (**Table 1**). Human Serpins also contain some tumor suppressor genes, including A5 (124), A11 (52), and B5 (125), which may inhibit tumor growth by inhibiting tumor metabolism.

**Table 1:** Target protease and expression of human serpins.

Clade	Serpin gene name	known aliases	Target protease	Cell and tissue expression	The function in psoriasis	Regulation in the epidermal barrier
A	SerpinA1	Antitrypsin	Inhibition of neutrophil elastase (21)	Liver, bone marrow, the lymphocytic and monocytic cells in lymphoid tissue, the Paneth cells of the gut, neutrophils (21, 27)	Loss of function caused by mutations in SerpinA1 activates IL-36α, which contributes to the development of GPP (28–31).	
	SerpinA2		Probable pseudogene	Testes, leukocytes (32)		
	SerpinA3	Antichymotrypsin(ACT)	Inhibition of chymotrypsin, cathepsin G (33, 34)	Liver, gall bladder, brain, prostate, testis, pancreas (34, 35)	Loss of function caused by mutations in SerpinA3 activates IL-36γ, which contributes to the development of GPP (36–39)	
	SerpinA4	kallistatin (PI4)	Inhibition of kallikrein (40)	Liver, gall bladder (40)		
	SerpinA5	Protein C inhibitor (PCI), plasminogen activator inhibitor-3 (PAI-3, PAI3)	Inhibition of active protein C, various plasminogen activators, kallikreins, thrombin (41)	Testis, adrenal, liver, gall bladder, kidney, endometrium (35, 42, 43)		
	SerpinA6	corticosteroid binding globulin(CBG)	non-inhibitory protein	Liver (44), brain (45), gall bladder, kidney (35).		
	SerpinA7	thyroxin-binding globulin (TBG)	non-inhibitory protein (46)	Liver (35)		
	SerpinA8	angiotensinogen (AGT)	non-inhibitory protein	Liver (47), brain, gall bladder, heart (35),skin (48)	Its gene polymorphism is associated with plaque psoriasis (17), but the exact mechanism has not been studied.	
	SerpinA9	Centerin (GCET1)	Inhibition of trypsin, thrombin, plasmin (49)	Lymph node, appendix, skin and germinal center B-cells in secondary lymphoid organs (35, 49, 50).		
	SerpinA10	PZI; ZPI	Inhibition of coagulation factor Xa and Xia (51)	Liver (35)		
	SerpinA11		Accelerating the degradation of urokinase-type Plasminogen Activator(uPA) (52)	Liver (35)		
	SerpinA12	Vaspin	Inhibition of kallikrein 7 (KLK7) (53) and 14 (KLK14) (54)	Skin (35), keratinocyte (55)	SerpinA12 is significantly reduced in the lesional skin of psoriasis patients. Decreased expression of SerpinA12 led to increased keratinocyte inflammation and decreased differentiation, and enhanced communication between keratinocytes and immune cells (55–57)	Reduced expression of SerpinA12 leads to decreased expression of the desmosomal proteins, the cornified envelope proteins, and keratins (55) that results in reduced impaired skin barrier.
B	SerpinB1	leucocyte elastase inhibitor (LEI); monocyte/neutrophil elastase inhibitor M/NEI;	Inhibition of neutrophil elastase, cathepsin G and proteinase-3 (58, 59), human granzyme H (60)	Macrophages and neutrophils (58), Bone marrow, esophagus, duodenum, small intestine (35)	SerpinB1 is elevated in lesional skin of psoriasis patients. SerpinB1 may inhibit the occurrence and development of psoriasis by inhibiting the formation of neutrophil NET (58) and limiting the undesirable proliferation of lymphocytes with the Th17 phenotype (61).	
	SerpinB2	plasminogen activator inhibitor-2(PAI-2)	Inhibition of urokinase plasminogen activator (uPA) (62)	Esophagus, skin, placenta, bone marrow (35), monocytes and macrophages (63), keratinocytes (64), fibroblasts (65) and eosinophils (66).	SerpinB2 is elevated in lesional skin of psoriasis patients. SerpinB2 can inhibit the proliferation of human keratinocytes (62). SerpinB2 depletion enhance the chemotaxis and immune response of immune cells by increasing inflammatory chemokines (67–70).	SerpinB2 deficiency was found to cause stratum corneum defect (64, 71)
	SerpinB3	SCCA1	Inhibition of cathepsin L, S, K and papain (15)	Esophagus, urinary bladder, skin (35), immune cells and many mucosal cells (15)	SerpinB3 expression is elevated in the skin lesions of GPP patients with SerpinB3 mutation (72). SerpinB3-derived protein Pso p27, an autoantigen in psoriasis, increases inflammation and promotes migration of immune cells. The increase in expression of SerpinB3 in the skin can promote inflammation and poor differentiation of keratinocyte (73–77)	SerpinB3 has been shown to cause epidermal barrier dysfunction (78)
	SerpinB4	SCCA2	Inhibition of chymase, Cathepsin G, Granzyme M (15)	Esophagus, urinary bladder, skin, appendix (35)	SerpinB4-derived protein Pso p27, an autoantigen in psoriasis, increases inflammation and promotes migration of immune cells The increase in expression of SerpinB4 in the skin can promote inflammation, poor differentiation of keratinocyte (73–77).	SerpinB4 has been shown to cause epidermal barrier dysfunction (78)
	SerpinB5	PI5; maspin	non-inhibitory protein	Esophagus, skin, urinary bladder, small intestine (35)	As an autoantigen of an autoimmune response induced by streptococcus, is the target of an enhanced T-cell response in psoriasis (79).	
	SerpinB6	PI6	Inhibition of cathepsin G (80), plasmin, thrombin and kallikrein-8) (81, 82)	blood cells, platelets, endothelial cells, keratinocytes and other epithelial cells (81), widely expressed in human tissues (35)		
	SerpinB7	Megsin		Mesangial cells (83), skin (84)	SerpinB7 deficiency inhibits keratinocyte differentiation and promote the expression of inflammatory mediators (85). However, as mentioned earlier, the expression of SerpinB7 in psoriatic lesions is reversed in different studies, and its regulation of inflammation depends on its expression level in psoriatic lesions, which needs to be confirmed by further research.	SerpinB7 deficiency can cause epidermal barrier dysfunction in IMQ-induced psoriasis like models (85)
	SerpinB8	PI8	Inhibition of furin (86)	was strongly expressed in the nuclei of squamous epithelium of mouth, pharynx, esophagus, and epidermis, and by the epithelial layer of skin appendages, particularly by more differentiated epithelial cells; by monocytes and by neuroendocrine cells in the pituitary gland, pancreas, and digestive tract (87)	SerpinB8 is a susceptibility gene for psoriasis (88), but the exact mechanism has not been studied.	
	SerpinB9	PI9	Inhibition of granzyme B (89)	accessory immune cells (including dendritic cells) (90)		
	SerpinB10	PI10, bomapin	Inhibition of thrombin and trypsin (91)	Bone marrow (35, 91)		
	SerpinB11	Epipin	noninhibitory intracellular protein (92)	Prostate, esophagus, urinary bladder (35)		
	SerpinB12	Yukopin	Inhibition of trypsin and plasmin (93)	the epithelial cells of most organs, including the respiratory tract, digestive system, and skin (35, 94)		SerpinB12 is a gene associated with epidermal permeability barrier, and its expression is decreased in atopic dermatitis model mice (95).
	SerpinB13	hurpin	Inhibition of cathepsin L (96)	Esophagus, skin, urinary bladder (35), blood, kidneys and saliva (97)		
C	SerpinC1	antithrombin III (ATIII)	Inhibition of coagulation proteases (98)	Liver (35)		
D	SerpinD1	heparin cofactor II	Inhibition of thrombin (21)	Liver (35)		
E	SerpinE1	plasminogen activator inhibitor-1(PAI1)	Inhibition of thrombin, tissue plasminogen activator (tPA), urokinase (uPA)[ (21)	Gall bladder, placenta, liver (35)		
	SerpinE2	glia-derived connexin (GDN)	Inhibition of Thrombin, urokinase (99), plasmin (100)	Placenta, brain, ovary (35)		
	SerpinE3		Not characterized	Testis, lymph node, skin, thyroid (35)		
F	SerpinF1	PEDF	non-inhibitory protein	Liver, gall bladder, fat, testis (35)		
	SerpinF2	A2AP; alpha2AP;	Inhibition of plasmin (101)	Liver, kidney (35)		
G	SerpinG1	complement I esterase inhibitor; C1 inhibitor(C1IN)	Inhibition of Complement I esterase (102)	Liver, gall bladder, ovary, skin (35).		
H	SerpinH1	HSP47	non-inhibitory protein; does not act as a protease inhibitor, but as a companion to collagen (103)	Placenta, endometrium, appendix, urinary bladder (42), blood, liver and heart (104).		
	SerpinI1	neuroserpin	Inhibition of tPA, uPA, and plasmin (5, 105)	Brain, kidney, testis (35)		
	SerpinI2	Myoepithelium-derived serine proteinase inhibitor(PI14); Pancpin	Inhibition of pancreatic chymotrypsin and elastase (106)	Pancreas (35)		

Some serpins are commonly expressed in various human tissues and cells and some serpins are expressed specifically in certain tissues, which we have summarized in **Table 1**. Serpins are abundant in expression and distribution in the skin. In the Clade A, SerpinA12 is mainly expressed in the skin and keratinocytes in the skin has been identified as a rich source of vaspin (serpinA12) (55). AGT is an important part of the renin-angiotensin system (RAS). Steckelings UM et al. found that the complete renin-angiotensin system exists in human skin (48). The majority of serpins containing(B1-8, B12, B13) in Clade B are expressed in the skin (35). SerpinB2 is expressed in keratinocytes of the skin and one study suggested that SerpinB2 (cross-linked to the cornified envelope) was present in the stratum corneum (71). In skin, SerpinB3 is expressed in the spinous and granular layers of normal epithelium (126). In normal skin, SerpinB13 expression is mainly confined to the basal layer, while in diseased skin, it is mainly distributed in the outermost layers of granular and upper spinous layers (127). In other branches, SerpinE3 and SerpinG1 are also expressed in the skin (35).

## 3 Serpins regulate epidermal barrier and maintain skin homeostasis

The exposed epidermis is a laminated squamous epithelium composed of multiple layers of keratinocytes. Epidermis is the physical barrier of our human skin, protecting us from moisture loss and mechanical damage. It is complemented by a chemical barrier composed of antimicrobial peptides and proteins to protect the host from the surrounding microbiota (16). Profilaggrin metabolism, the formation of cornified envelope, desmosomes, intercellular lipid lamellae and zonula occludens, desquamation play important roles in the integrity of the epidermal barrier (128–130). The differentiation of keratinocytes affects the formation of the cornified envelope that is an insoluble protein and lipid structure with barrier functional properties (131, 132). As mentioned earlier, many members of the serpin family are expressed in the skin, which play a an important role in regulating epidermal barrier and maintain skin homeostasis. Reduced expression of SerpinA12 leads to decreased expression of the desmosomal proteins, the cornified envelope proteins, and keratins (55) that results in reduced impaired skin barrier. As a predominantly expressed adipokine in the skin (55, 115), SerpinA12 may alter its expression or functional activity by inhibiting kallikrein 7 (KLK7), which KLK7 controls desquamation and is a key molecule in the maintenance of skin barrier function (53, 133–135). Reduced expression of SerpinA12 in keratinocyte has been reported to decreased expression of the desmosomal proteins, cornified envelope proteins, and keratins (55) which are proteins that maintain the integrity of the epidermal barrier. The decrease of SerpinA12 expression may weaken the inhibition of KLK7 activity. Abnormal KLK activity impairs epidermal barrier function (136). So we hypothesized that reduced SerpinA12 expression may lead to impaired epidermal barrier by inhibiting KLK7 activity.

SerpinB2 is considered to be one of the precursors of the envelope and is involved in the formation of the cornified envelope (64, 71). SerpinB2 deficiency was found to cause stratum corneum defect and more susceptible to topical application of inflammatory agents. The function of SerpinB2 in keratinocytes is to protect the stratum corneum from proteolysis by inhibiting urokinase, thereby maintaining integrity of the stratum corneum and its barrier effect, especially during skin inflammation (71). Epidermis with high SerpinB3 expression level increase sensitivity to barrier destruction by external stimuli, suggesting that SerpinB3 plays an important role in inducing epidermal barrier disruption (137). As a molecule elevated in both psoriasis and atopic dermatitis patients, SerpinB3/4 has been shown to cause epidermal barrier dysfunction in experimental mouse models of atopic dermatitis (78). As a common target of SerpinB3 and SerpinB13, cathepsin L is the elusive enzyme that processes and activates transglutaminase 3(TGM3) (138). The TGM is involved in formation of cornified envelope in the epidermis and plays an important role in epidermal barrier (138). Both SerpinB3 and SerpinB13 are highly expressed in psoriasis lesions (18, 139). Psoriasis showed impaired epidermal barrier function (140). So we hypothesize that increased SerpinB3/B13 expression suppress TGM3 activation by inhibiting cathepsin L activity, thereby affecting cross-linking of the stratum corneum and resulting in impaired skin barrier. SerpinB7 deficiency can cause epidermal barrier dysfunction in IMQ-induced psoriasis like models (85). SerpinB12 is a gene associated with epidermal permeability barrier, and its expression is decreased in atopic dermatitis model mice (95). Impaired epidermal function is a significant feature of atopic dermatitis (78). However, it is unclear whether SerpinB12 is a key molecule and specific mechanism that regulates impaired epidermal barrier function in atopic dermatitis, and further research is needed.

## 4 Pathogenic roles of serpins in psoriasis

At present, the majority of serpins focus on the pathogenesis of psoriasis vulgaris. SerpinA12 and SerpinG1 is significantly elevated in the serum of patients with psoriatic arthritis (141, 142), but its specific mechanism of action in psoriatic arthritis has not been reported. Next, we summarized the pathogenic role of serpins in psoriasis.

### 4.1 Modulation of keratinocyte hyper-proliferation and differentiation

Although the clinical features of psoriasis differ in different psoriasis types, most types of psoriasis lesions are characterized by erythema, thickening, and scale (13). Thickening and scale are associated with excessive proliferation of keratinocytes (143). Parakeratosis, the persistence of nuclei in the stratum corneum, is one of the histopathological features of psoriasis (144) and is related to excessive proliferation and poor differentiation of keratinocytes (145). Poor keratinocyte differentiation also promotes impaired skin barrier function (146).

SerpinA12 expression is significantly reduced in the lesional skin of psoriasis patients (115). Reduced expression of SerpinA12 in psoriatic keratinocytes leads to decreased expression of differentiation-related genes (Loricrin, Involucin, Keratin1 and 10) (55), which cause poor differentiation. SerpinB2 and B3 are highly expressed in the epidermis of psoriatic patients (18, 117). SerpinB2 can inhibit the proliferation of human keratinocytes (62). SCCA1 is always found in parakeratotic epidermis (137), which suggests that it may be related to the proliferation and differentiation of keratinocytes. SerpinB7 and SerpinB13 are both keratinocyte differentiation regulators (147, 148). As mentioned earlier, SerpinB13 is highly expressed in psoriasis lesions (139). Overexpression of SerpoinB13 in keratinocytes can decrease UV-induced apoptosis by inhibiting cathepsin L (96). SerpinB7 has been identified as a skin-specific endogenous protease inhibitor and a novel psoriatic-associated gene that is highly expressed in keratinocytes of psoriatic patients and imiquimod-induced psoriatic lesions in mice (85, 149). However, one study found that SerpinB7 expression was down-regulated in psoriatic skin lesions, and its expression level is negatively correlated with the severity of the patient’s disease (150). Zheng H et al. (85) found that SerpinB7 deficiency down-regulates the expression of differentiation-related genes (Keratin 10, Loricrin, Filaggrin, Involucrin) and increased keratinocyte proliferation by decreasing the calcium ion influx (85). The expression of SerpinB7 in psoriatic lesions is controversial. Whether SerpinB7 has a positive or negative effect on the pathogenesis of psoriasis depends on its expression level, which needs to be further verified. SerpinB8 is a susceptibility gene for psoriasis (88), but the exact mechanism has not been studied. SerpinB8 inhibit its amidolytic activity by forming an SDS stable complex with furin (151). Furin is a prohormone convertase involved in inflammation, prohormone processing, and extracellular matrix remodeling (86). Furin is involved in the cleavage of the precursor protein profilaggrin into the monomer filaggrin, and filaggrin plays an important role in keratinocyte differentiation (130). So we hypothesized that SerpinB8 might be involved in keratinocyte differentiation by regulating furin activity, but this has not been studied.

### 4.2 Contribution to immune response and inflammation

Psoriasis vulgaris is a chronic inflammatory skin disease caused by the interaction of keratinocytes and immune cells (145), and is thought to involve self-perpetuating inflammatory mechanisms via the IL-23/Th17 axis (13). Munro microabscesses filled with neutrophils in psoriatic skin lesions are considered to be typical histopathological markers of psoriasis. Neutrophils communicate and interact with antigen-presenting cells and lymphocytes at the site of inflammation (152, 153). Circulating neutrophils are recruited to the site of inflammation after inflammatory signals or bacterial induction in skin, and are then activated to produce respiratory bursts that produce large amounts of ROS, degranulation and neutrophil extracellular trap(NET) production, which contributes to the pathogenesis of psoriasis (154). In patients with psoriasis, neutrophils are pre-activated and form a NET in psoriatic skin lesions (155, 156) NET promote keratinocyte to secrete high levels of proinflammatory factors (LCN2, IL-36γ, CXCL8, and CXCL1) by activating TLR4/IL-36R crossers and the downstream MyD88/NF-κB signaling pathway (156). These chemokines include LCN2, CXCL8, and CXCL1, which in turn promote neutrophil migration to the skin (154). NET chromatin in psoriatic plaques, together with the antimicrobial peptide LL-37 released by keratinocytes, often stimulates the secretion of inflammatory factors such as IL-12 and IL-23 by dendritic cells (157, 158), which then activates the differentiation of T cells into Th1, Th17 and Th22, and promotes them to secrete of TNF-α and IFN-γ, IL-17, IL-22, respectively (13). IL-17 secreted by Th17 cells is the main effector of psoriasis. IL-17 not only activates neutrophils but also promotes the proliferation of keratinocytes and the production of inflammatory chemokines(CXCL1, CXCL8, CCL20, TNF) by binding to IL-17 receptors. CCL20 and TNF are chemokines of T cells and dendritic cells, respectively (154), which in turn leads to a T cell immune response. IL-23 plays an significant role in the survival and proliferation of Th17 and Th22, and the Th17 pathway mediated by IL-23 is considered to be the main pathway in psoriasis (13). The release of LCN2 and MPO during neutrophil degranulation exacerbate skin inflammation in psoriasis by participating in the activation of neutrophils (154, 159). ROS produced during respiratory bursts activate mitogen-activated protein kinase (MAPK), nuclear factor-κB (NF-κB), or Janus kinase-signal transducer and activator of transcription proteins (JAK-STAT) (JAK-STAT) associated inflammatory pathways (160), which induce the proliferation and inflammation of keratinocytes (47, 161). In general, neutrophils play an important role in the initiation and maintenance of psoriasis through ROS production, degranulation and NET formation.

Like SerpinB2, B3, SerpinB1 is elevated in lesional skin of psoriasis patients (18, 67, 117, 162). SerpinB1 is also found to be abundant in the cytoplasm and granules of neutrophils (163). SerpinB1, a potent inhibitor of NE, has recently been shown to limit neutrophil extracellular trap(NET) production (58) and has also been found to limit the undesirable proliferation of lymphocytes with the Th17 phenotype (61),. The expression levels of SerpinB2 in the psoriasis-involved skin are positively correlated with the severity of psoriasis (67, 117). There is evidence that RNA silencing of SerpinB2 in keratinocytes leads to upregulation of IL-8, CXCL5 and CCL5 and increased neutrophil migration (117). Expressions of IL-8, CXCL5 and CCL5 are all increased in psoriatic lesions, where CXCL5 and IL-8 are neutrophil chemokines that drive neutrophils to the skin, and CCL5 is a chemokine of Th1 cells that produce IFNγ (68–70). IFNγ, in turn, stimulates the activation of dendritic cells, which triggers a T cell immune response (13). These studies suggest that SerpinB2 depletion is involved in the pathogenesis of psoriasis by increasing inflammatory chemokines to enhance the chemotaxis and immune response of immune cells. As an anti-inflammatory miRNAs, miR-146a/b can indirectly iinhibit SerpinB2 expression by targeting IL-1 receptor-associated kinase 1(IRAK1) and caspase recruitment domaincontaining protein 10(CARD10) in human primary keratinocytes (117). The expression of MiR-146a/b in psoriatic lesions was negatively correlated with the expression of SerpinB2. MiR-146a/b collaborates with serpinB2 in keratinocytes to regulate inflammation in psoriasis. These studies show that SerpinB1and B2 may play a negative regulatory role in the pathogenesis of psoriasis. The literature shows that pro-inflammatory cytokines secreted by infiltrating immune cells in the skin can induce SERPINB3 to be highly expressed in psoriatic skin (72). The SerpinB3/4-derived protein Pso p27, an autoantigen in psoriasis and other chronic inflammatory diseases, is only expressed in the psoriasis involved skin, not in uninvolved skin. In psoriasis, SerpinB3/4 are synthesized by skin fibroblasts and keratinocytes, and then taken up by mast cells to form Pso p27 through cleavage of chymase in mast cells (18). Pso p27 can bind to either IgG or complement factor C1q in the skin lesions of psoriasis patients to form and activate immune complexes (73, 74), which boosts the skin’s immune response. TEA domain family member 4 (TEAD4), as a transcription factor (75), is overexpressed in keratinocytes of psoriasis (76). Silencing of TEAD4 in keratinocytes leads to reduced expression of SerpinB3/4, which suggests TEAD4 can transcriptionally regulate the expression of SerpinB3/4 (76). Decreased expression of SerpinB3/4 inhibits the expression of immune cell chemokine (CXCL1,5,8) in keratinocytes (76). TEAD4 silencing in keratinocytes reduces T migration and the secretion of IL-17 and IL-22 in T cells, which in turn reduces the secretion of CXCL1,5,8 in keratinocytes (76). One Study have shown that SerpinB3/4 stimulates keratinocytes to produce inflammatory chemokines and promote CD4 +T cell migration by activating the NF-κB signaling pathway (77). These results suggest that TEAD4 may increase the crosstalk of keratinocyte and T cell by enhancing the expression of SerpinB3/4, thereby promoting the cytokine secretion of keratinocyte and T cell, and T cell migration. SerpinB5, as an autoantigen of an autoimmune response induced by streptococcus, is the target of an enhanced T-cell response in psoriasis (79).

SerpinB7 deficiency significantly increased the expression of chemokines (TNF-α, IL-1b, IL-23, CXCL2 and IFN-γ), neutrophil markers Ly6G, and antimicrobial peptide S100A8, thereby exacerbating skin inflammation (85). SerpinB7 deficiency promote the expression of inflammatory mediators by decreasing the calcium ion influx (85) However, as mentioned earlier, the expression of SerpinB7 in psoriatic lesions is reversed in different studies, and its regulation of inflammation depends on its expression level in psoriatic lesions, which needs to be confirmed by further research.

**Figure 2:**
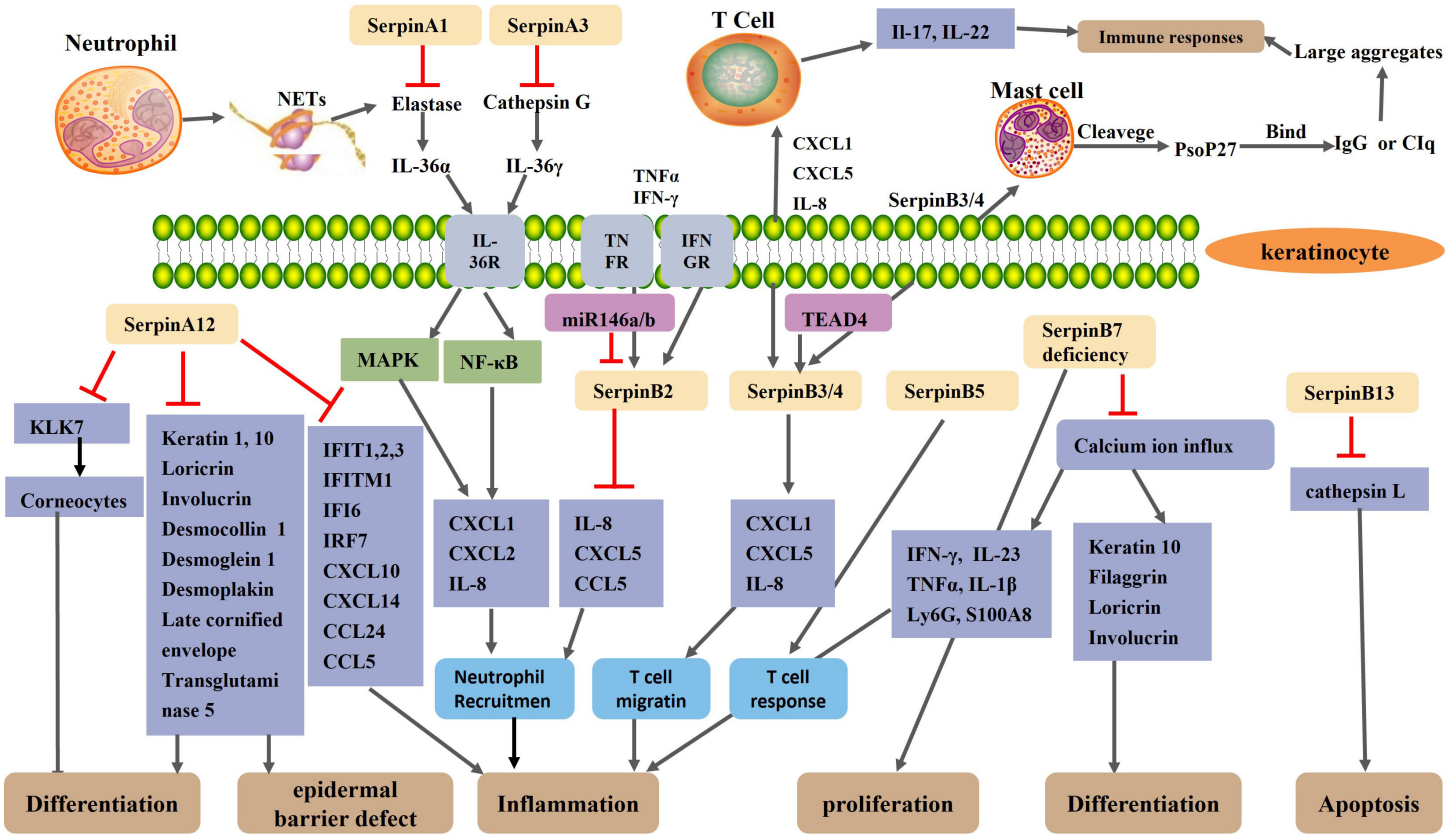
Schematic representation of the regulatory mechanism of Serpin protein in psoriasis. A neutrophil extracellular trap(NET) structure formed after neutrophil necrosis or apoptosis exposes neutrophil elastase and cathepsin G to the extracellular environment. SerpinA1 and SerpinA3 inhibit neutrophil elastase and cathepsin G, respectively. Normally, elastase and cathepsin G activate IL-36α and IL-36γ, respectively, thereby activating the MAPK and NF-κB signaling pathways of keratinocytes, resulting in increased expression of CXCL1, CXCL2, and IL-8 and increased recruitment of neutrophils. SerpinA12, as an anti-inflammatory factor, is reduced in the skin lesions of psoriasis patients. The reduction of SerpinA12 leads to an overactivation of KLK7 and thus an excessive loss of corneocytes, which impair epidermal barrier. Reduced expression of SerpinA12 in KC reduces keratinocyte differentiation and exacerbates inflammation by reducing the expression of differentiation-related genes and psoriasis related inflammatory gene. The expression of SerpinB2 in keratinocytes is up-regulated under the stimulation of the TNF-α and IFN-γ. As an anti-inflammatory miRNAs, MiR-146a/b collaborates with serpinB2 in keratinocytes to inhibit inflammation in psoriasis. SerpinB2 deficiency leads to upregulation of IL-8, CXCL5 and CCL5 and increased neutrophil migration. SerpinB3/B4 are taken up by mast cells to form Pso p27 through cleavage of chymoenzymes in mast cells. TEAD4 may modulate keratinocyte and T cell crosstalk by targeting SerpinB3/4, thereby affecting keratinocyte and T cell cytokine secretion and T cell migration. SerpinB5 is an autoantigen of an autoimmune response and is the target of an enhanced T-cell response in psoriasis. SerpinB7 deficiency significantly increased the expression of chemokines, neutrophil markers Ly6G, and antimicrobial peptide S100A8, thereby exacerbating skin inflammation. SerpinB7 deficiency inhibits keratinocyte differentiation and promotes the expression of inflammatory mediators by decreasing the calcium ion influx.

As mentioned earlier, SerpinA12 expression is significantly reduced in the lesional skin of psoriasis patients (115). Reduced expression of SerpinA12 in psoriatic keratinocytes leads to increased expression of the interferon-inducible (56, 57) and psoriasis related inflammatory genes (chemokine ligand 20, IL-6, IL-8, and S100 proteins) (55). Reduced expression of SerpinA12 in keratinocytes stimulated co-cultured dendritic cells, macrophages, monocytes, and neutrophils to secrete tumor necrosis factor-α, IL-1β, IL-6, IL-8, and monocyte chemotactic protein-1 (55). The reduced expression of SerpinA12 in human epidermis enhance the communication between keratinocytes and immune cells (55). Immunohistochemical studies have shown that the serine protease KLK7 and KLK14 is increased in psoriasis lesions compared to normal skin (164, 165). As the main target protease of SerpinA12, KLK7 controls the process of the activation of pro-inflammatory IL-1β and prochemerin, all of which are involved in the pathogenesis of psoriasis (133, 134, 166). KLK7 also controls the enzymatic processing of antimicrobial peptide precursors in the skin and regulates the function of antimicrobial peptides, which act as immunomodulators in psoriasis (167). Antimicrobial peptides, such as LL-37, proteins ADAMTSL5, K17, and hsp27, may act as autoantigens to promote differentiation of autoreactive lymphocytes and unleash chronic inflammatory responses (168). Uncontrolled activity of KLK7 can lead to psoriasis (169). SerpinA12 and KLK7 are co-located in the skin (134). Based on these results, we hypothesize that SerpinA12 inhibits KLK7 in normal skin, but decreased SerpinA12 expression in psoriatic lesions reduces the inhibition of KLK7 activity, thereby exacerbating the inflammatory response and immune response. But this requires further direct verification (**Figure 2**).

Transcriptome analysis showed that SerpinA8 (AGT) is one of the differentially expressed genes in mild psoriasis (17). As an inflammation gene, the gene polymorphism of SerpinA8 is associated with plaque psoriasis and a positive family history of psoriasis (20), however the specific mechanism of SerpinA8’s involvement in psoriasis has not been studied. SerpinA8 is the only precursor of all angiotensin peptides. As an important product of AGT, AngII not only plays an important role in the regulation of blood pressure, but also plays a dual role in the regulation of inflammation. AngII has the opposite effect by activating AT1R and AT2R, respectively. Ang II interacts with AT1R to play a pro-inflammatory role, while Ang II and AT2R play an anti-inflammatory role (47, 170). Preliminary studies have shown that keratinocytes have the ability to produce Ang II (and potentially other angiotensin) independently of the supply of systemic RAS components. Ang II can induce potent inflammation associated with IL-17 (47, 171). IL-17 is a major effector of psoriasis that activates the NF-κB signaling pathway (172). Ang II induces reactive oxygen species (ROS) production by stimulating NADPH oxidase (NOX) (47, 173). So we hypothesize that SerpinA8 gene polymorphisms induce inflammation and keratinocyte proliferation by regulating Ang II expression, and thus participate in the pathogenesis of psoriasis.

## 5 Involvement of serpins in generalized pustular psoriasis (GPP)

GPP is a severe form of psoriasis, which is characterized by large, visually visible pustules on the non-extremity skin, with or without systemic symptoms such as fever, neutrophilism, and elevated serum C-reactive protein levels (12).

Kantaputra P et al. (72) found that the skin of GPP patients with SerpinB3 mutation showed high expression of SerpinB3. The mutations in both SerpinA1 and SerpinA3 are likely to be predisposing risk factors of generalized pustular psoriasis (GPP) (19). A heterozygous missense mutation of SerpinA1 occurs in patients with GPP (19). SepinA1 can inhibit the activity of elastase in neutrophils (28), and it has been found that SepinA1 mutation can cause overactivity of elastase (29). The overactivity of neutrophil elastase can promote the activation of IL-36α, and the activated IL-36α can enhance the ability of keratinocytes to produce chemokines (30). The inhibitory capacity of SerpinA1 is reduced in symptom-free and in those with stationary lesions patients with psoriasis (31). Barszcz D et al. (31) suggested that the abnormal function of SerpinA1 may be related to the pathogenesis of psoriasis. Frey S, et al. found a rare loss of function mutation in the entire reactive center loop of SerpinA3 in a small number of patients with GPP (36, 37). Liu Y, et al. (38) identified four novel mutations in SerpinA3 in seventy children with GPP and demonstrated that three of these mutations lead to loss of function of ACT (the protein encoded by SerpinA3), thereby reduce the inhibition of ACT on Cathepsin G. Cathepsin G (a protease produced by infiltrating neutrophils in the epidermis) can activate IL-36γ in the epidermis of patients with GPP, and activated IL-36γ induces an increased ability of keratinocytes to produce chemokines (CXCL1, CXCL2 and CXCL8) and enhance recruitment of neutrophils, thereby exacerbating skin inflammation (30, 39). In the skin of GPP patients, the expression and activity of IL-36α and IL-36γ is elevated in keratinocytes located around neutrophil microabscesses (30). Therefore, we speculate that similar to SerpinA3, SerpinA1 is involved in the pathogenesis of GPP through loss-of function mutations, but further studies are needed to confirm it (**Figure 2**).

## 6 The potential therapeutic strategies targeting serpins in psoriasis

In summary, the changes in the expression of some serpins in the skin participate in the pathogenesis of psoriasis by regulating the inflammation, differentiation and interaction with immune cells of keratinocytes. When the expression of some serpins increases, their transcriptional regulators that inhibit serpin expression or corresponding antibodies can be developed to reduce the expression of serpins. When the expression of some serpins reduces, psoriasis can be treated with its recombinant protein. For example, in an animal model of psoriasis, the application of recombinant vaspin (SerpinA12) reduced infiltration by myeloid cells into the skin (55).

Mutations and polymorphisms of some serpins are also involved in the pathogenesis of psoriasis, and we can use their downstream product related antagonists to treat psoriasis. For example, losartan act as an angiotensin receptor (AT1R) antagonist, attenuates imiquimod-induced psoriasis-like inflammation (171).

## 7 Conclusions

Serpins play an complex role in regulation of the epidermal barrier and the development of psoriasis. Some serpins, including SerpinA12, SerpinB2/3//7 play multiple roles in skin barrier function and pathogenesis of psoriasis. The decrease in the expression of SerpinA12, SerpinB7 deficiency and increase in expression of SerpinB3/4 in the skin can promote inflammation, poor differentiation of keratinocyte and damaged skin barrier. Pso p27, derived from SerpinB3/B4, is an autoantigen that can enhanced immune response in psoriasis. However, whether SerpinB3 is participating in the pathogenesis of psoriasis by itself or by hydrolyzing into Pso p27 has not been investigated. The expression of SerpinB7 in psoriasis is controversial, which requires further studies to confirm. SerpinB2 plays a role in maintaining epidermal barrier integrity and inhibiting keratinocyte proliferation. Some anti-inflammatory serpins(SerpinB1, B2) are highly expressed in psoriasis, which may be the body’s response to the development of the disease. Some serpins(SerpinA1, A3, B3) cause GPP through a genetic mutation that triggers skin inflammation. AGT(SerpinA8) and SerpinB8 are susceptibility gene for psoriasis, but the specific mechanism has not been studied. Based on the relevant literatures, we summarized the possible mechanism of AGT and SerpinB8 to provide reference for future research. The underlying mechanisms of serpins have not been fully elucidated and needs to be further explored. The study of serpins in the pathogenesis of psoriasis may provide a novel therapeutic target for the treatment of psoriasis.
